# Cross-linked chitosan aerogel modified with Pd(II)/phthalocyanine: Synthesis, characterization, and catalytic application

**DOI:** 10.1038/s41598-019-50021-6

**Published:** 2019-09-25

**Authors:** Amal Al-Azmi, Sajjad Keshipour

**Affiliations:** 1 Chemistry Department, Kuwait University, P. O. Box 5969, Safat, 13060 Kuwait; 2 Department of Nanochemistry, Nanotechnology Research Center, Urmia University, Urmia, Iran

**Keywords:** Chemistry, Catalysis

## Abstract

Palladium(II) phthalocyanine (PdPc) tetrasulfonate was chemically bonded to an amine moiety of chitosan aerogel. The reaction was promoted by the transformation of sulfonic acid groups of PdPc to sulfonyl chloride, which is highly active for amination. The porous composite showed good catalytic activity in the oxidation reaction of some alkylarenes, aliphatic and benzylic alcohols, and cyclohexanol. High conversions and excellent selectivities were obtained for the solvent-free reactions under aerobic conditions at 80 °C during 24 h. While many oxidation reactions have been reported catalysed with palladium phthalocyanine, this is the first reported oxidation of alkylarenes via this catalyst. The organometallic compound is applicable as a heterogeneous catalyst having high chemical stability with recyclability up to six times.

## Introduction

The harmful effects of hazardous materials encourage organic researchers to develop new procedures using biocompatible catalysts to supress pollutant effects of chemical processes. Accordingly, biodegradable materials are increasingly utilized in organic transformations. For example, many heterogeneous catalysts have been synthesized using biocompatible materials such as cellulose and chitosan^1,2^. Chitosan, as a chiral polyamine, has attracted the researcher’s interest due to its important role in many applications^3–6^. This naturally-based polymer shows good flexibility, insolubility in many solvents, and affinity for most metal ions^7,8^. Recently, the porous form of chitosan, known as chitosan aerogel (CA), has been used extensively in biomedical applications, catalysis, removal of pollutants, and insulating materials due to its high aspect ratio^9–16^. In addition, CA can be used as a support for catalysts because the amine groups on the backbone of the polymer allow to easy chemical modifications, and the high surface area of CA affords a high number of available sites for reactions^17–20^.

Oxidation reactions of organic substrates are considered one of the main organic transmutations and are powerful strategies for achieving valuable compounds from natural materials. Many studies have been carried out to promote them according to green chemistry principles. Aerobic catalytic oxidations have gained green chemistry credentials because they are free from toxic oxidizing agents which avoid from the hazardous waste formation^21,22^. Metalophthalocyanines (MPcs) are known, as potent catalysts for oxidation reactions, obtained industrial interest arising from their high performance, easy preparation, inherent nontoxicity, recovery possibility, and recyclability^23^. Pcs of transition metals such as Co(II), Fe(II), and Cu(II) have been employed in the oxidation reactions of various organic substrates. While Pd(II)-Pc is an efficient catalyst for the C-C coupling in Suzuki reaction and reduction of nitroarenes^24^, its catalytic activity for oxidation reactions is not well investigated. Oxidations of trichlorophenol^25^, 1,3-diphenylisobenzofuran^26^, 4-nitrophenol^27^, and chlorophenol^28^ are some examples of reactions promoted using PdPc. To the best of our knowledge, PdPc catalytic activity has not been extended to the oxidation of alkylarenes.

In continuation of our efforts to modify biopolymers such as chitosan with Pd-*D*-penicillamine and Au(III)-dimercaprol^29,30^, herein we modified CA with PdPc tetrasulfonate to achieve a biocompatible catalyst for the oxidation of alkylarenes. Loading PdPc on CA has at least the following two advantages: (1) distribution of the catalyst on a high-surface-area support sophistically increases the number of active sites for the reaction, and accordingly, the required catalyst amount is decreased; and (2) heterogeneity of the prepared catalyst facilitates the recovery of the catalyst.

## Results and Discussion

**Figure 1:**
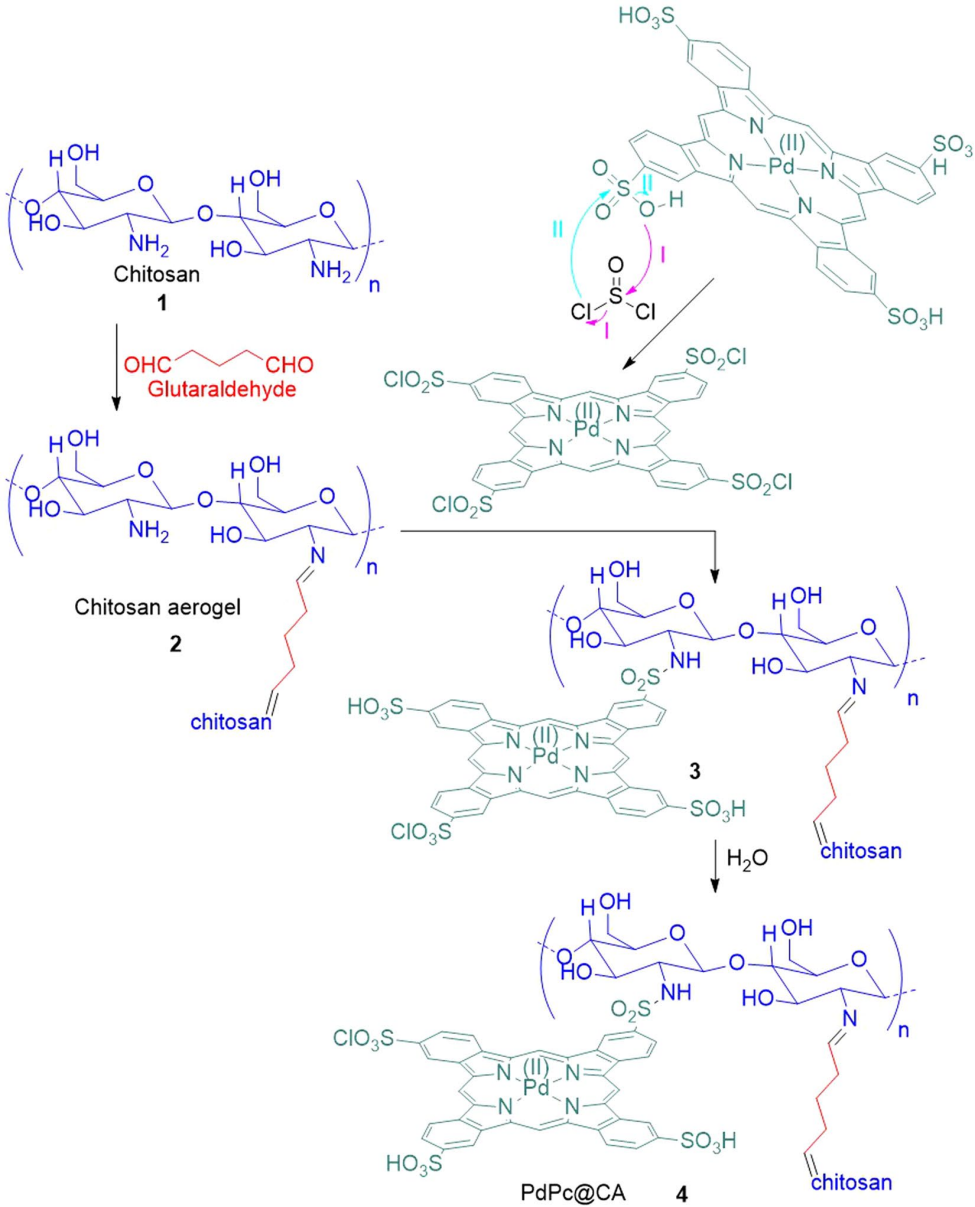
General protocol for the synthesis of PdPc@CA.

Anchoring the catalyst on a high aspect ratio support is one of the strategies for increasing the catalyst’s efficiency in low amounts, which produces a heterogeneous catalyst with recyclability potential. In this study, PdPc tetrasulfonate as a catalyst was bonded to CA through sulfonic acid groups. For this purpose, sulfonic acid groups of tetrasulfonated PdPc were activated by thionyl chloride to achieve sulfonyl chloride functionality, which under amidation reaction with CA produces PdPc@CA (Fig. 1).

### FT-IR spectra

**Figure 2:**
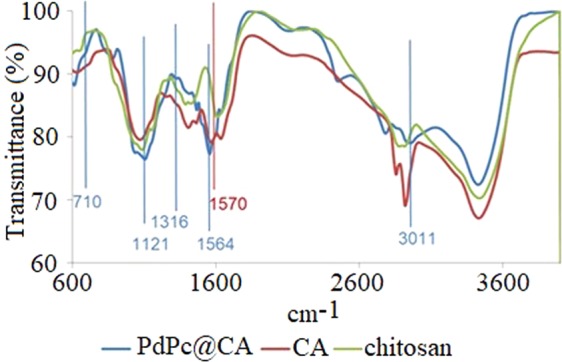
FT-IR spectra CA (**A**), PdPc (**B**), and PdPc@CA (**C**).

FT-IR spectra confirmed success of the employed modification process for the synthesis of PdPc@CA. The appearance of a new absorption band in the CA spectrum at 1570 cm^−1^ for imine moiety indicated crosslinking the chitosan by glutaraldehyde to produce CA. Depositing PdPc tetrasulfonate on CA led to new absorption bands on the PdPc@CA spectrum, such as 3010 cm^−1^ for aromatic C – H, 1564 cm^−1^ for imine bind, 1121 and 1316 cm^−1^ for S = O bind, and 710 cm^−1^ for new S – N bind (Fig. 2)^31^.

### X-Ray Diffraction (XRD) pattern of PdPc@CA

**Figure 3:**
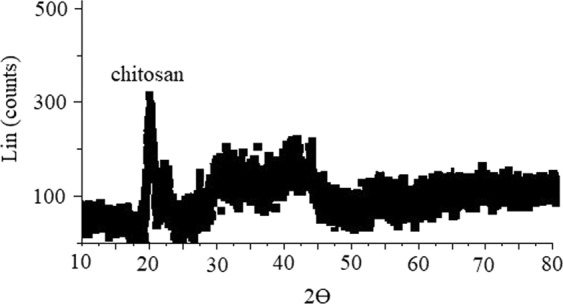
XRD pattern of PdPc@CA.

The XRD pattern of PdPc@CA showed main peaks at 2Ɵ = 22.7, 25.9, 28.3, and 43.4; the peak at 2Ɵ = 22.7 belongs to chitosan^29^, and the other peaks are related to phthalocyanine (Fig. 3)^32^. Broadening of the peaks indicate amorphous structure of PdPc@CA.

### CHNS elemental analysis and Flame Atomic Absorption Spectroscopy (FAAS) of PdPc@CA

Quantitative determination of PdPc on CA requires FAAS and CHNS analysis. The CHNS analysis of PdPc@CA revealed that C was 42.71%; H, 6.82%; N, 8.57%; and S, 2.32%. Since each PdPc has four sulfonyl groups, the mol% of PdPc is 1/4 of S. Therefore, 100 g of PdPc@CA has 0.0181 mol of PdPc. The Pd loading on the catalyst was also determined to be 1.9 g (0.018 mol) per 100 g of the catalyst according to FAAS analysis.

Significantly, CHNS analysis and FAAS result confirm each other.

1 $${\rm{mol}}\,{\rm{of}}\,{\rm{S}}=2.32/32=0.0725$$

2 $${\rm{mol}}\,{\rm{of}}\,{\rm{PdPc}}=({\rm{mol}}\,{\rm{S}})/4=0.0181$$

### X-ray Photoelectron spectroscopy (XPS) of PdPc@CA

**Figure 4:**
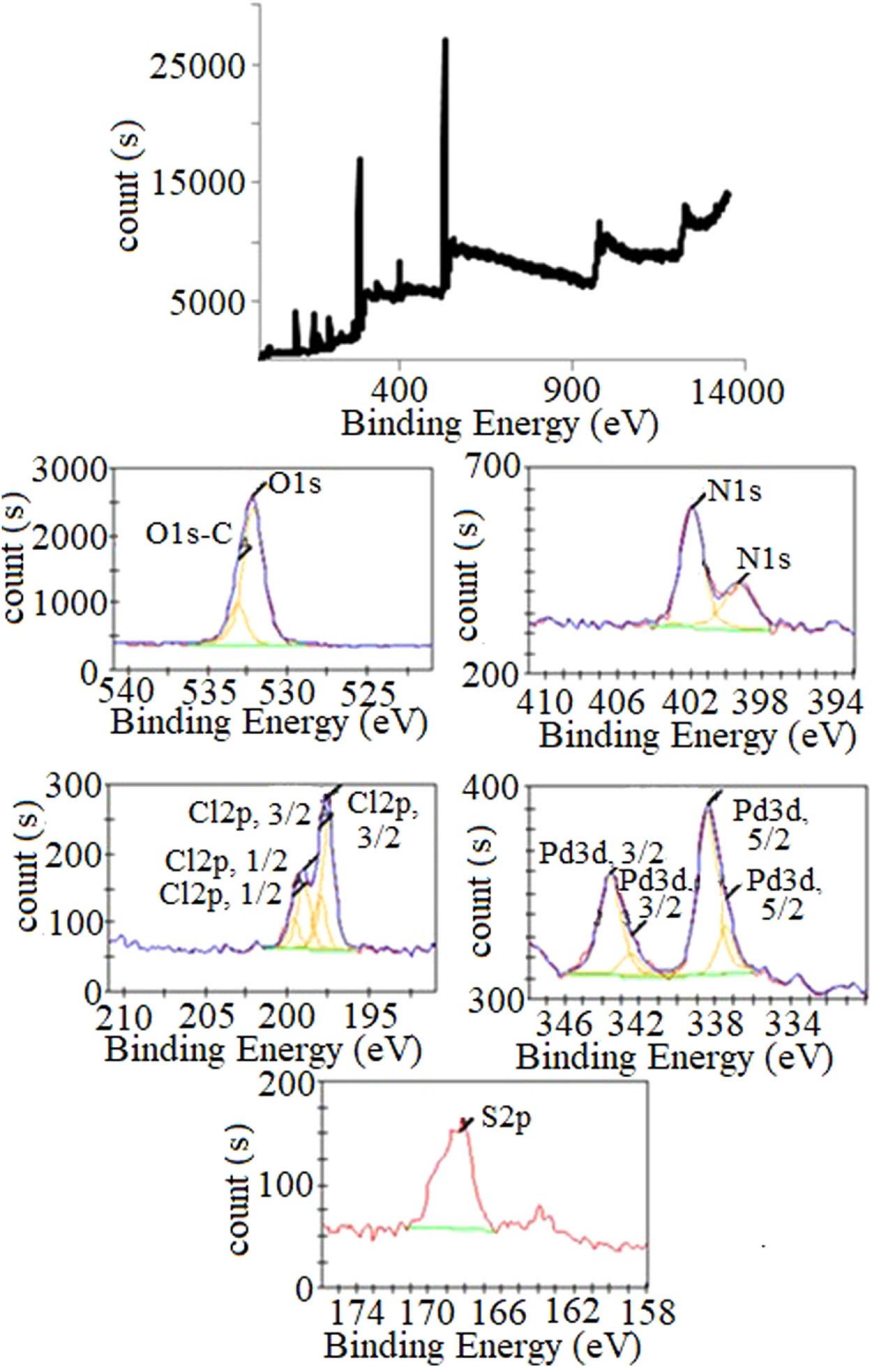
XPS spectrum of PdPc@CA.

To confirm the oxidation numbers of atoms on PdPc@CA, XPS analysis was performed (Fig. 4) which approved loading of PdPc on CA. The expansion of the spectrum around 400 eV indicated a doublet peak for nitrogens. Nitrogens of phthalocyanines with sp^2^ hybrid peaked at 399.1 eV, while the nitrogens of sulfonamides with sp^3^ hybrid peaked at 401.2 eV. The peak at 401.2 eV obviously supports the formation of the N – S bind during the modification reaction^33,34^. Pd(II) showed a doublet peak at 338.4 and 343.9 eV, confirming the existence of PdPc on CA^35^. A peak at 168.3 eV is attributed to S of the SO_3_H group^36^. The doublet intrinsic to the S peak together with the observation of Cl peaks at 197.3 and 199.2 eV indicated that some of the sulfonyl chloride groups were intact on the catalyst.

### Scanning electron microscope (SEM) images of chitosan and PdPc@CA

**Figure 5:**
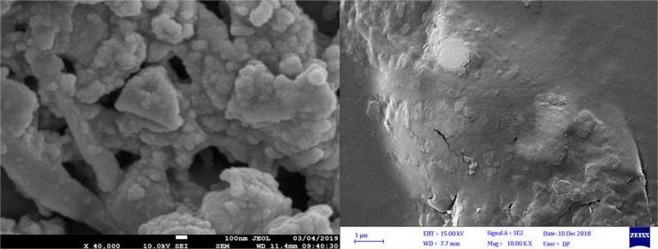
SEM image for PdPc@CA (left) and chitosan (right).

The SEM image of PdPc@CA demonstrates a porous network attributed to the CA. The procedure employed for the preparation of CA was solving the chitosan to obtain a gel and then removing the solvent to generate fine particles of chitosan in a porous network. As seen in Fig. 5, a decrease in the particle size has been performed successfully, which is more clearly observable in the comparison SEM image of PdPc@CA with chitosan.

### Brunauer-Emmett-Teller (BET) analyses of chitosan and PdPc@CA

**Figure 6:**
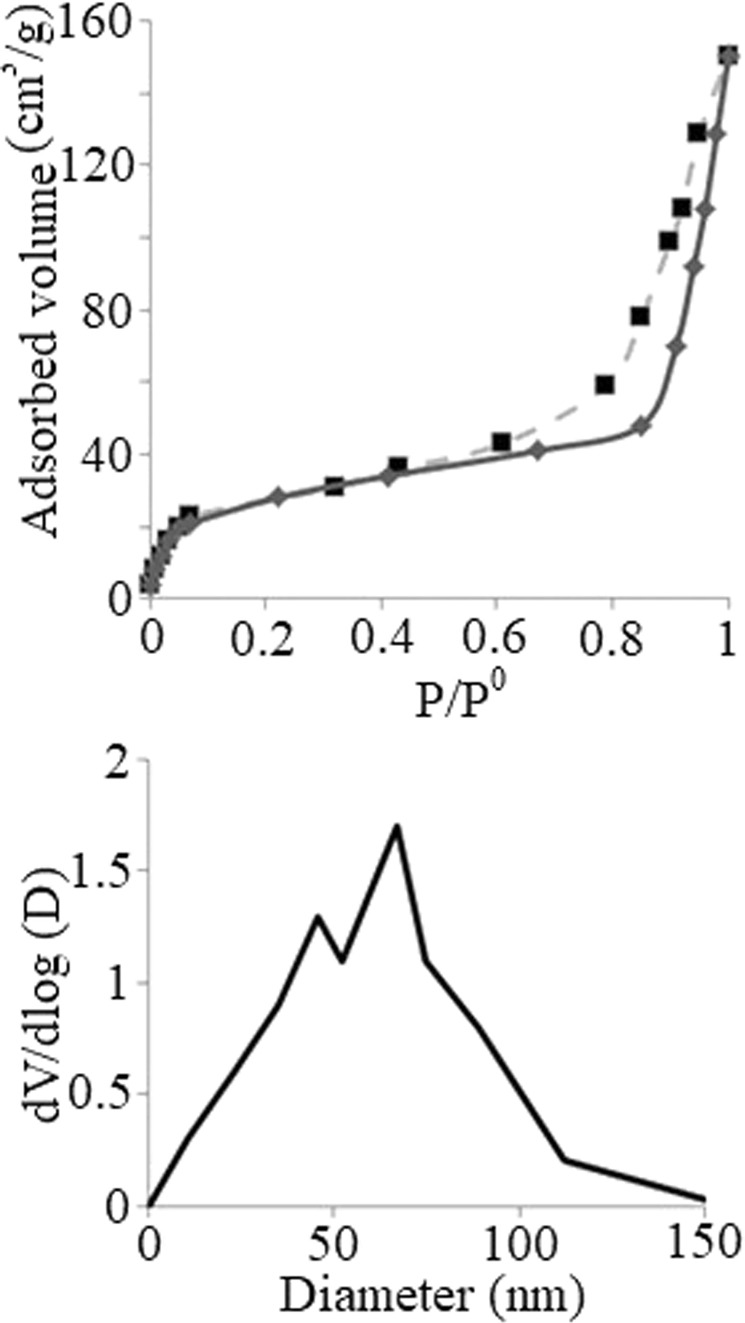
N_2_ adsorption-desorption isotherms for PdPc@CA and size distribution diagram.

BET analysis of PdPc@CA with nitrogen (N_2_) adsorption/desorption at 77 K shows that the surface area for PdPc@CA is 52.14 m^2^/g (Fig. 6). A significant difference was observed between the surface area of PdPc@CA and employed chitosan with 0.53 m^2^/g surface area. Increasing the aspect ratio of PdPc@CA compared to chitosan let us to have a catalyst with highly available reaction sites. Nitrogen sorption analysis of PdPc@CA revealed the presence of macropores, which is also shown by a pore size diagram with a maximum size of 67 nm.

### Thermal gravimetric analysis (TGA) of PdPc@CA

**Figure 7:**
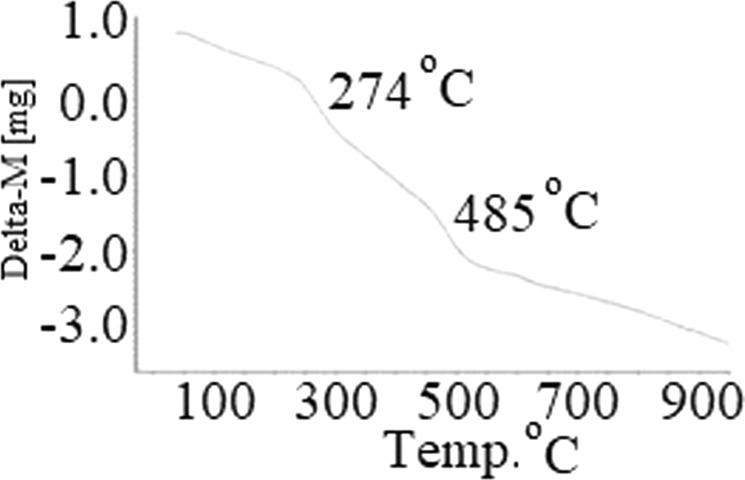
TGA curve of PdPc@CA.

The thermal stability of PdPc@CA was investigated with TGA in the air. Good thermal stability was observed with decomposition above 274 °C due to the degradation of the polymeric structure into small units with some degasification (Fig. 7)^37^. The second decomposition point was observed above 485 °C. This result is related to the decomposition of new small units formed at the previous decomposition temperature^37^.

### Catalytic examination of PdPc@CA

The catalytic activity of PdPc@CA was investigated in the oxidation reaction of alkylarenes. At first, the oxidation of ethylbenzene was selected as the model reaction, with an O_2_ balloon as the oxidant in the presence of PdPc@CA. The reaction needed 0.036 mmol (0.2 g) of PdPc@CA under solvent-free conditions at 80 °C to produce high yields (Table 1). Gas Chromatography (GC) analysis of the product showed acetophenone as the product with 73% conversion and 100% selectivity. Decrease in the catalyst amount from 0.036 to 0.018 mmol declined the conversion from 73% to 66% and its increase to 0.054 mmol did not improve the conversion sophistically (Table 1, entries 1–3). The reaction gave 73% conversion at 80 °C and it was decreased significantly at 70 °C to 58%, while increasing temperature to 90 °C did not afford higher yields compared to 80 °C. A control experiment under N_2_ atmosphere indicated that the reaction needs O_2_ for performing (Table 1, entry 6). Among various solvents screened for the oxidation reaction of ethylbenzene, acetonitrile produced the highest yields compared to other solvents. However, solvent-free conditions were the best for the oxidation reaction because they preserved the green chemistry credentials.

**Table 1:** Optimization of the reaction conditions for the oxidation of ethylbenzene^a^.

Entry	Catalyst (mmol)	Temp. °C	Solvent	Conversion (%)
1	0.018	80	—	66
2	0.036	80	—	73
3	0.054	80	—	74
4	0.036	70	—	58
5	0.036	90	—	73
6^b^	0.036	80	—	0
7	0.036	80	H_2_O	52
8	0.036	reflux	EtOH	54
9	0.036	reflux	CH_3_CN	71
10	0.036	reflux	CH_2_Cl_2_	38
11	0.036	80	PhCH_3_	69

^a^Reaction conditions: ethylbenzene (2 ml), PdPc@CA, O_2_ balloon, solvent (5 ml), 24 h. ^b^In the absence of O_2_ and under N_2_ atmosphere.

The oxidation reaction was developed using various alkylarenes, such as propylbenzene, 4-ethylphenol, 1,2,3,4-tetrahydronaphtalene, and diphenylmethane (Table 2). High conversions and excellent selectivities were obtained for the oxidation reactions. The reaction also was examined for divers’ aliphatic and aromatic alcohols. PdPc@CA transformed liquid aromatic alcohols to the corresponding aldehydes in high yields under solvent-free conditions in 24 h at 80 °C. For solid alcohols such as 4-nitrobenzyl alcohol and 4-chlorobenzyl alcohol, acetonitrile has been used as the solvent under reflux conditions to produce high yields. Selectivities for aromatic alcohols reached 92–98% with benzoic acid derivatives as the byproducts. PdPc oxidized aliphatic alcohols and cyclohexanol to the corresponding aldehydes or ketones effectively with 100% selectivity for the secondary alcohols. The oxidation reaction for allylalcohol was failed because of missing large amounts of the substrate during the reaction. This problem is attributed to the low boiling point of allylalcohol.

**Table 2:** Oxidation of various substrates with PdPc@CA^a^.

Entry	Substrate	Product	Conversion (%)	Selectivity (%)
1			73	100
2			71	100
3			71	100
4			67	100
5			78	98
6			72	96
7^b^			74	95
8^b^			72	92
9			59	96
10			53	97
11			64	100
12			67	100

^a^Reaction conditions: substrate (2 ml), PdPc@CA (0.036 mmol), O_2_ balloon, 80 °C, 24 h. ^b^In CH_3_CN (5 ml) under reflux conditions.

### Proposed mechanism

**Figure 8:**
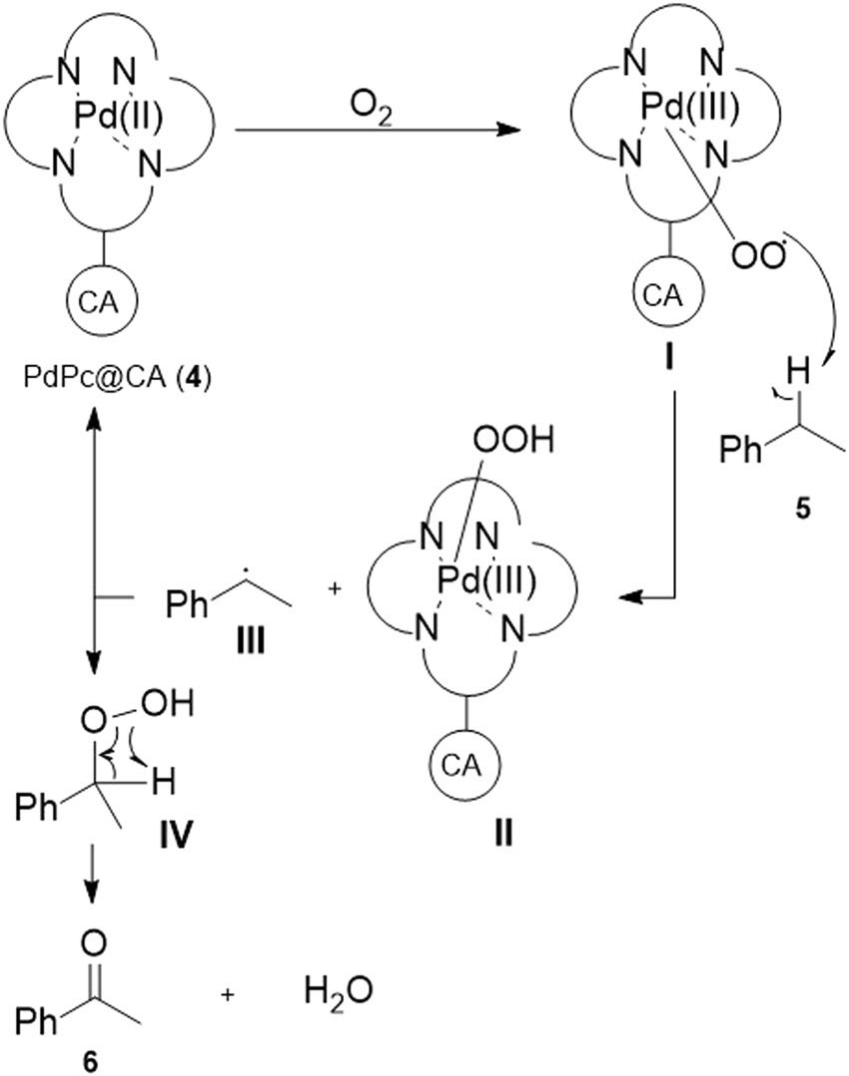
Proposed mechanism.

The mechanism of ethylbenzene oxidation using PdPc@CA catalyst is conceivable as Fig. 8 according ability of O_2_ complexation with Pd species. Anchoring O_2_ to PdPc@CA gives intermediate **I** which under proton abstraction from ethylbenezene (**5**) gives intermediates **II** and **III**. In continue, intermediate **III** takes hydroperoxide released from the specie **II** and was transformed to compound **IV**. (1-Hydroperoxyethyl)benzene (**IV**) under a rearrangement produce acetophenone (**6**) and H_2_O.

### PdPc@CA leaching study

Potential PdPc leaching into the mixture of the benzyl alcohol oxidation reaction was also analysed with FAAS. For this purpose, the sample was taken through a syringe filter during the heterogeneous oxidation reaction of benzyl alcohol and was dissolved in HNO_3_ for 1 h. The FAAS analysis of the sample showed that the Pd concentration in the reaction mixture was less than the detection limit (less than 9 μg/L). This result indicates that virtually no Pd leaches from PdPc@CA into the mixture. This procedure was also performed for the benzyl alcohol oxidation reaction in the presence of CA mixed with PdPc (0.036 mmol), obtaining a Pd concentration of 337 μg/L. Therefore, PdPc@CA has great chemical stability compared to PdPc mixed with CA.

### Recyclability of PdPc@CA

**Figure 9:**
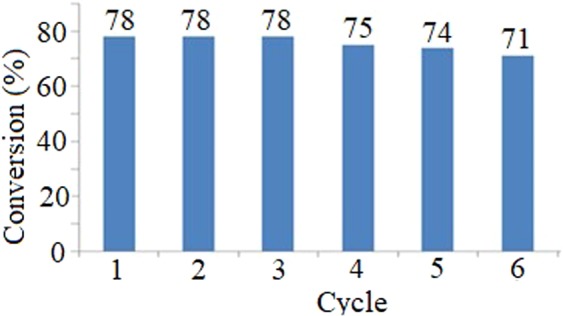
Successive trials using recoverable PdPc@CA for the oxidation of benzyl alcohol.

The recyclability of PdPc@CA was investigated in the oxidation reaction of benzyl alcohol. After carrying out the reaction, the catalyst was separated via filtration as a brown solid, washed with acetone (2 × 5 ml), and reused. Only a minor decrease in the reaction yield was observed after six repetitive cycles for this reaction (Fig. 9). To investigate the catalyst stability, the amount of Pd on the catalyst was determined before use and after six runs, obtaining 0.18 mmol per 1 g catalyst for both of them.

### Comparison of the results

A comparison was performed between the results of benzyl alcohol oxidation with PdPc@CA and some previously supported metallophthalocyanines such as FePc^38^, CoPc^32^, and CuPc (Table 3)^39^. FePc and CoPc transformed benzyl alcohol to benzaldehyde under milder reaction conditions in a shorter time compared to PdPc@CA. The oxidation reaction with CuPc gave high selectivity in a short reaction time, similar to the reaction with CoPc. However, PdPc@CA utility in the reaction under green conditions such as aerobic oxidation through a solvent-free approach showed both high yield and selectivity. While the oxidation of phenol has been reported with PdPc^27^, this work extended the application of PdPc to the oxidation of various alcohols and alkylarenes. Recyclability of the catalyst is another important characteristic of PdPc@CA which led to the minimizing waste. The catalytic activity of PdPc@CA was compared with that of PdPc and PdPc@CA. PdPc as the catalyst (0.036 mmol) gave much lower conversions than PdPc@CA, yet with higher selectivity. This result shows that the reaction can be performed with a low catalyst amount when the catalyst is dispersed on a support. Moreover, PdPc@C afforded low yields compared to PdPc@CA because the high surface area of CA provided more available reaction sites.

**Table 3:** Comparison of the results for the oxidation of benzyl alcohol.

Entry	Catalyst	Solvent	Oxidant	Time (h)	Temp. °C	Selectivity (%)	Yield (%)
1	Polyflouro/FePc	—	TBHP	3	50	81	97
2	CoPc@Cellulose	*o*-xylene	O_2_	8.5	r.t.	100	83
3	CuPc@MCM-41	dioxane	TBHP	4	100	100	36
4	PdPc@CA	—	O_2_	24	80	89	78
5	PdPc	—	O_2_	24	80	94	29
6	PdPc@C	—	O_2_	24	80	88	62

## Conclusion

In conclusion, PdPc was immobilized on chitosan aerogel by chemical bonding. This organometallic compound was applied as a heterogeneous catalyst for the oxidation of alkylarenes, aliphatic alcohols, benzyl alcohols, and cyclohexanol with high yields and excellent selectivities. All the reactions gave the corresponding aldehydes or ketones under aerobic oxidation with O_2_. Most of the reactions were solvent free. The catalyst proved recyclable and no catalyst leaching to the reaction mixture was observed. This is evidence that the new catalyst is chemically stable.

## Materials and Methods

All reagents were purchased from Merck or Aldrich and used without further purification. Chitosan (80–90% deacetylated, 2000 MW) was obtained from Golden-shell Biochemical Co., Ltd. (Zhejiang, China). GC High Resolution Gas Chromatograph Mass Spectrometer was carried out using Thermo Scientific. SEM images were prepared with a JEOL JSM 7001F. Pd determination was carried out on a FAAS (Shimadzu 105 model AA-680 atomic absorption spectrometer) with a hollow cathode lamp. The elemental analysis was performed with an Elementar Analysensysteme GmbH VarioEL. BET surface area was measured by nitrogen (N_2_) adsorption/desorption at 77 K using a QuadraSorb SI surface area analyzer after degassing the samples at 100 °C for 10 h. High resolution was carried out using a Thermo Scientific Mass Spectrometer. XRD pattern was obtained using a Bruker D8 ADVANCE X-ray diffractometer with a Cu-K_α_ radiation source (λ = 1.5406 Å) operating at 40 kV, 40 mA, and a scanning range of 10–80° 2θ, with a 2θ scan step of 0.015° and a step time of 0.2 second. Fourier transform infrared spectroscopy (FTIR) was used to characterize different functional groups of the composite using a Jasco 6300 FTIR instrument in the range of 600–4000 cm^−1^. XPS spectra were recorded on a Thermo ESCALAB 250 Xi using monochromatic Al K_α_ radiation (1486.6 eV) with a spot size of 850 µm. The spectra acquisition and processing were carried out using Thermo Avantage software. The sample was stuck on the sample holder using a double-sided carbon tape and then introduced into the preparation chamber and was degassed until the proper vacuum was achieved. Then it was transferred into the analysis chamber, where the vacuum was 9–10 mbar. The analysis was carried out using the following parameters: pass energy of 20 eV, dwell time of 50 ms, and step size of 0.1 eV. Selectivity was calculated as: (peak area of the desired product/sum of the peak areas for desired and by-product) × 100.

### Preparation of PdPc@CA

#### Synthesis of CA

Chitosan (2.00 g) was dissolved in an acetic acid solution (50 ml, 1.00 vol %) containing glutaraldehyde (2.00 ml). The mixture was stirred vigorously for 2 min to produce a wet gel within 10 min, which then aged for 24 h. Then, unreacted acetic acid and aldehyde in the wet gel were exchanged with absolute ethanol at 25 °C, and the aerogel (2.19 g) was obtained by freeze-drying at −40 °C^40^.

### Preparation of PdPc@CA

Thionyl chloride (1.00 g) was poured into a vessel containing PdPc tetrasulfonate (2.00 g)/DMF (10 ml), and the mixture was stirred at 50 °C under N_2_ atmosphere for 4 h. Then, the mixture was added dropwise to a balloon containing CA (5.00 g)/DMF (5 ml), and the stirring was continued for another 24 h at room temperature. Finally, the reaction was quenched by pouring H_2_O (10 ml), and the residue was filtered off, washed with acetone (3 × 5 ml), and dried in an oven at 50 °C to give a dark brown powder (5.11 g).

### Determination of Pd(II) on PdPc@CA using Flame Atomic Absorption Spectroscopy (FAAS)

PdPc@CA (0.05 g) was added to a mixture of HCl:HNO_3_ (3:1) (10 ml) and sonicated for 3 h. The mixture was filtered, and the total volume of the filtrate was increased to about 20 ml with addition of distilled water. The final solution was then aspirated into the flame of the AAS against the blank prepared with CA. The Pd(II) concentration was obtained using a calibration curve prepared with Pd solution standards.

### Typical procedure for the oxidation of ethylbenzene

In a typical procedure, ethylbenzene (2 ml) was added to a round-bottom flask containing the catalyst (0.036 mmol or 0.2 g), and the mixture was stirred at room temperature under O_2_ atmosphere created by a O_2_ balloon. The progress of the reaction was monitored by thin layer chromatography (TLC). Upon completion, the catalyst was separated via filtration. Then, the mixture was analysed with GC.

## Supplementary information

GC graph
