# Supplementary material for: Cross-linked chitosan aerogel modified with Pd(II)/phthalocyanine: Synthesis, characterization, and catalytic application
Source: Sci Rep. 2019 Sep 25;9:13849. doi: 10.1038/s41598-019-50021-6 (PMC6761259; doi:10.1038/s41598-019-50021-6)

Supporting Information:

Cross-linked chitosan aerogel modified with Pd(II)/phthalocyanine: Synthesis, characterization and catalytic application

Amal Al-Azmi,^a*^ Sajjad Keshipour^b^

^a^ Chemistry Department, Kuwait University, P. O. Box 5969, Safat 13060, Kuwait.

* Correspondent e-mail: [amalrchem@gmail.com](mailto:amalrchem@gmail.com), [amal.alazemi@ku.edu.kw](mailto:amal.alazemi@ku.edu.kw).

^b^ Department of Nanochemistry, Nanotechnology Research Center, Urmia University, Urmia, Iran.

GC spectra:


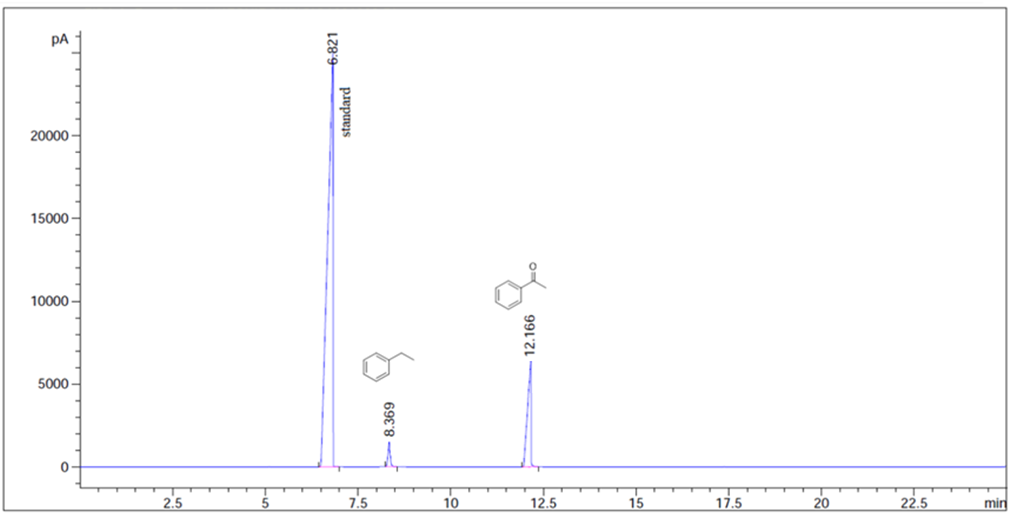


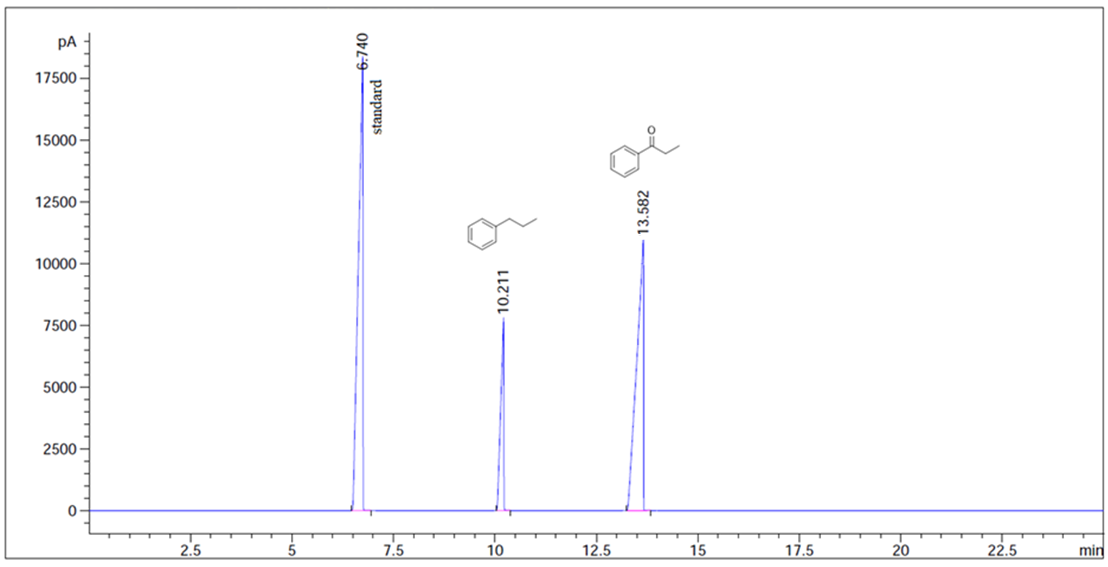


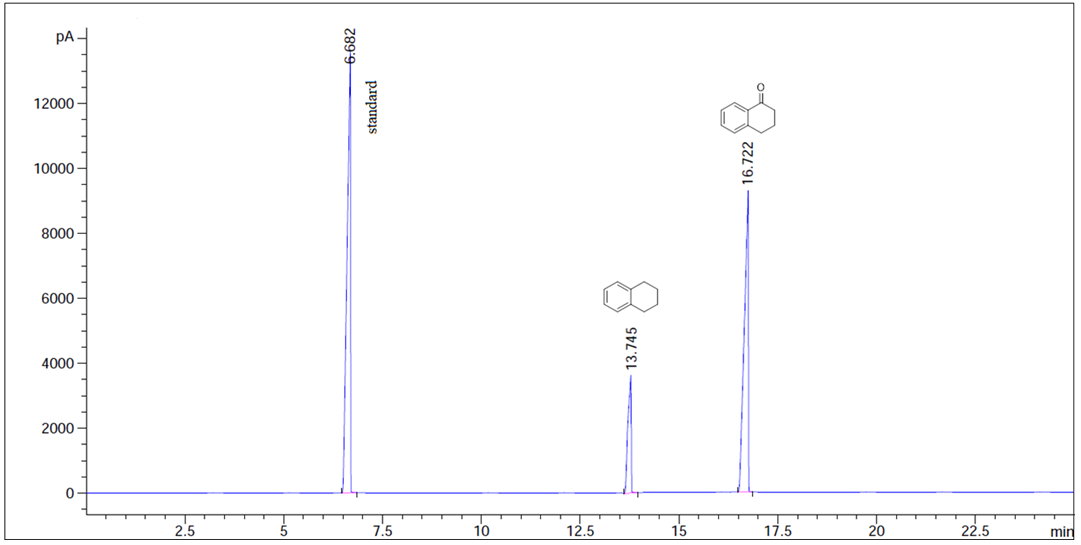


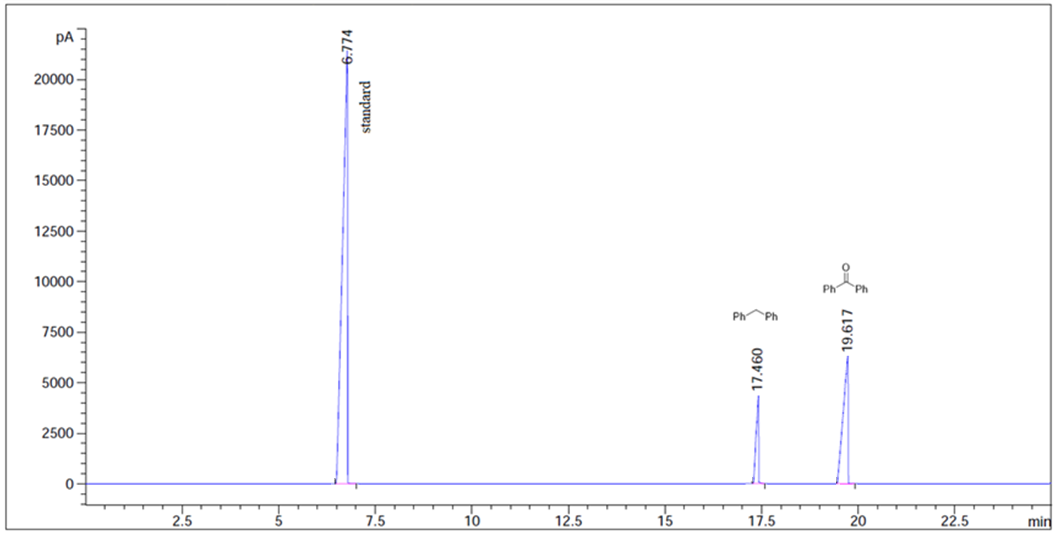


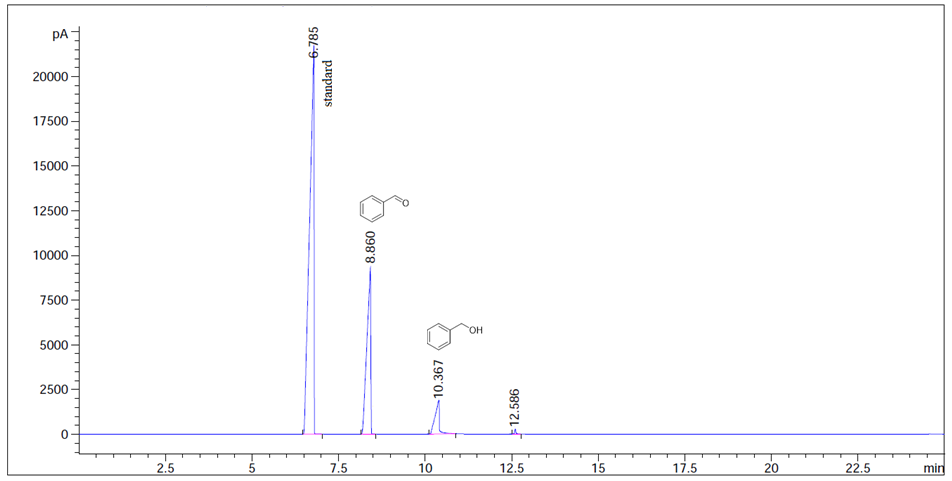


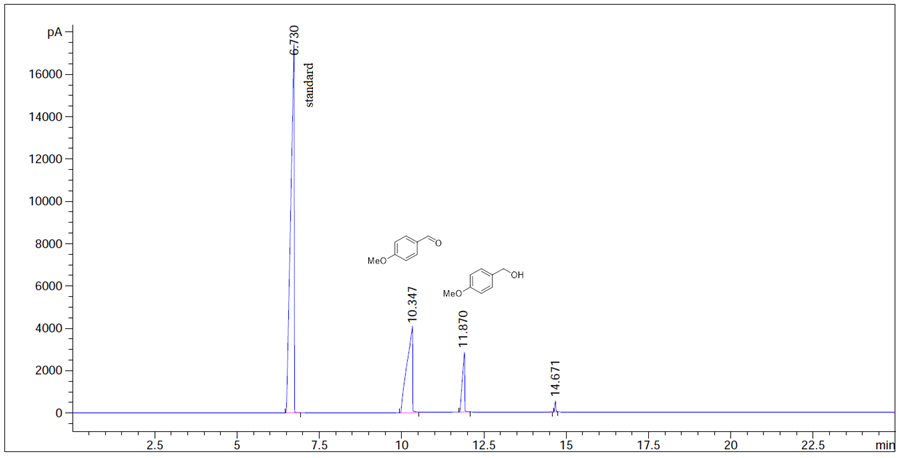


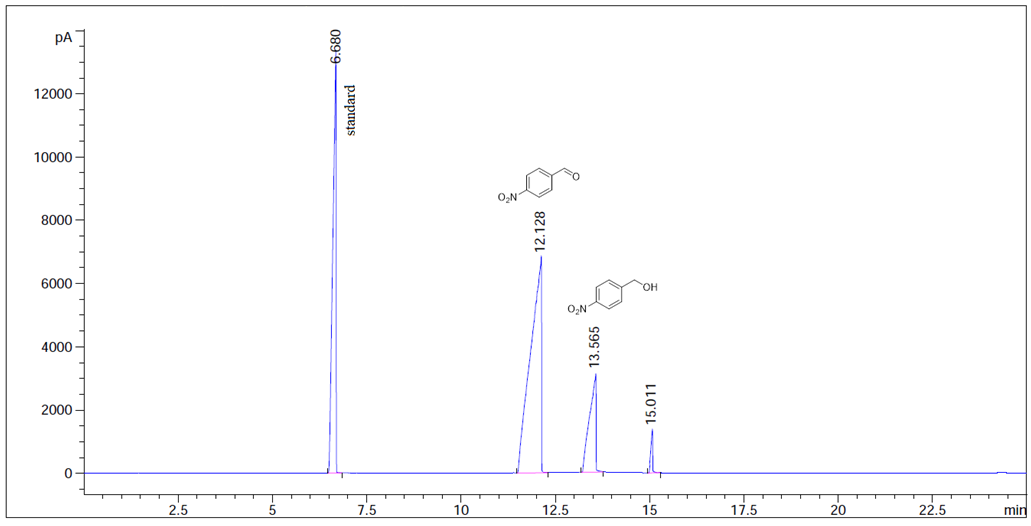


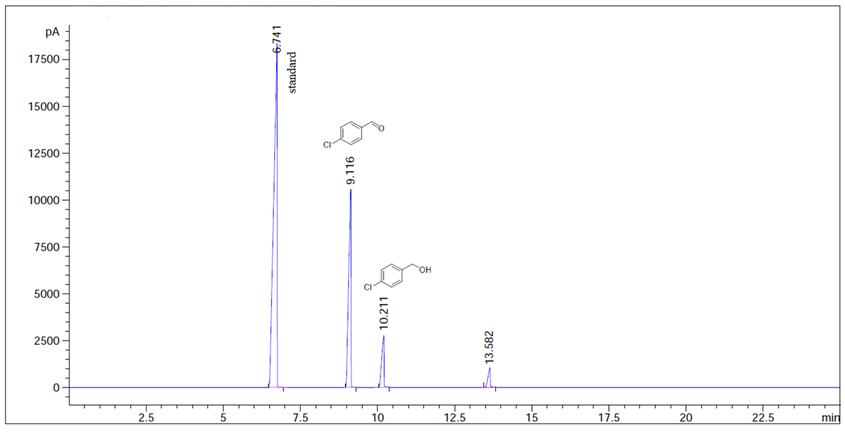


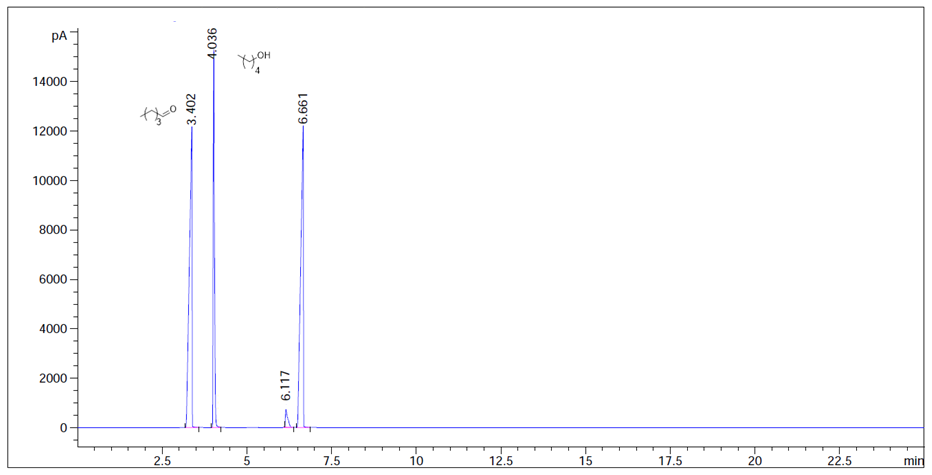


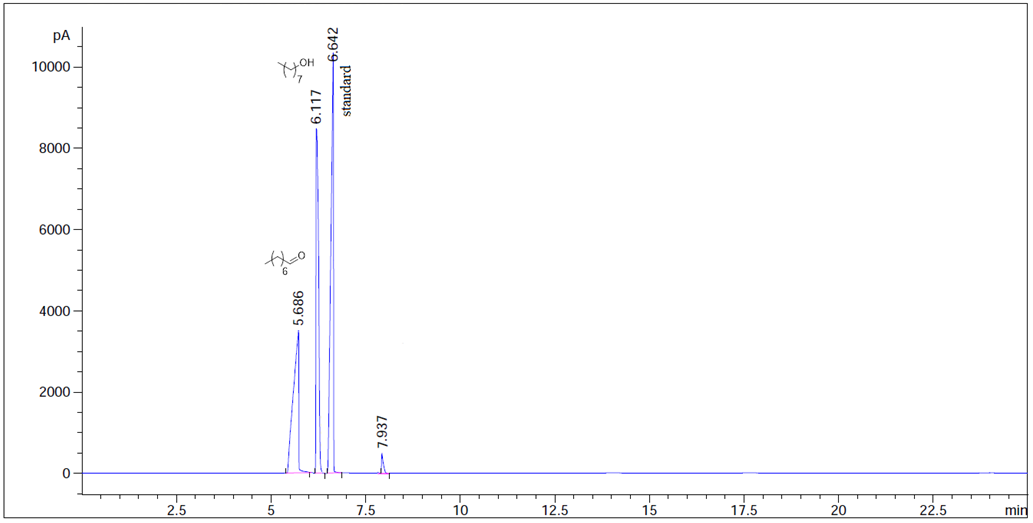


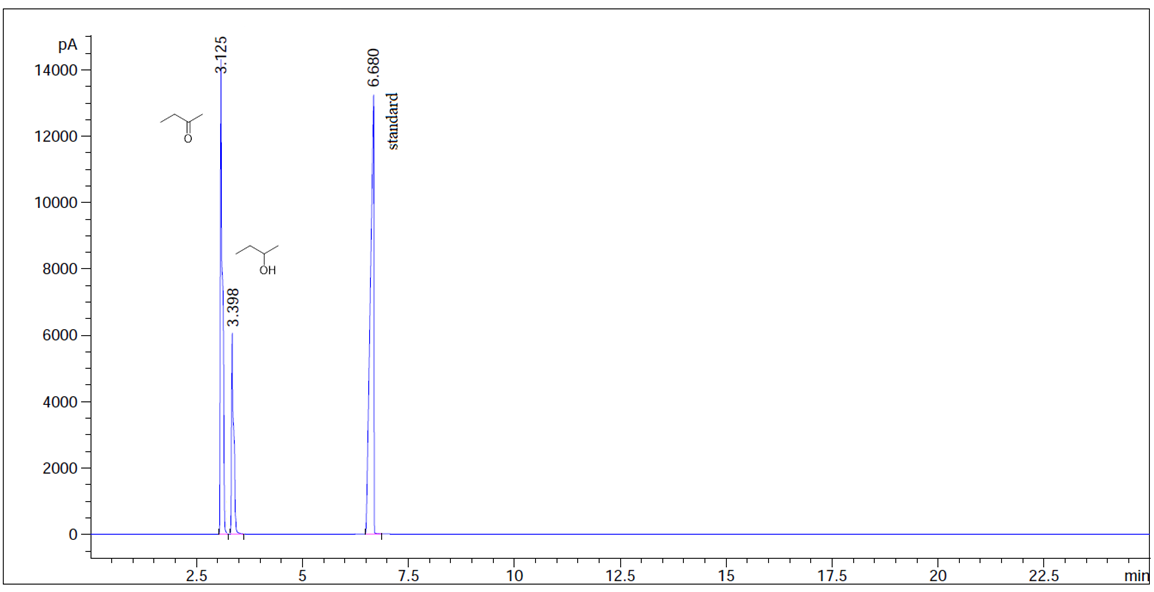


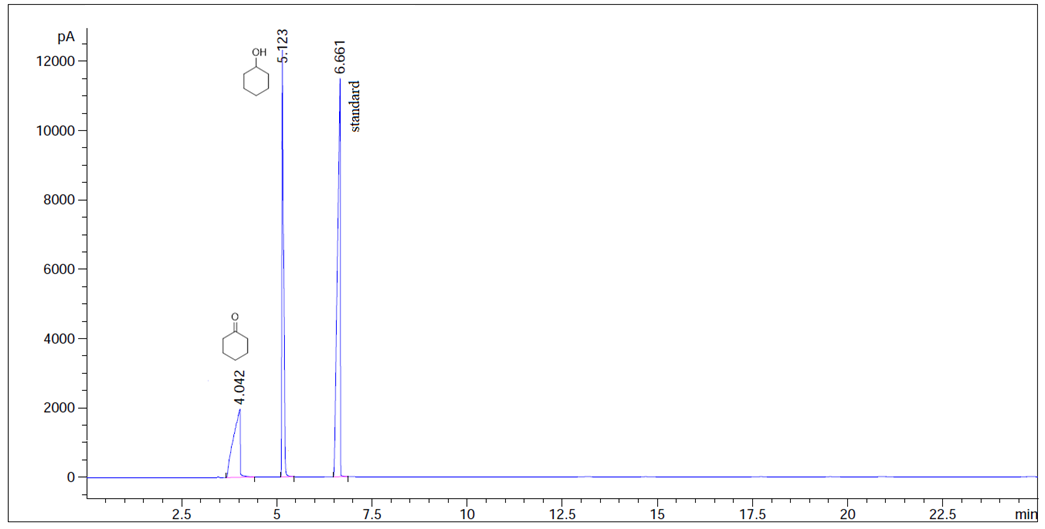

Supplement: Supplementary file 1 — GC graph [file 41598_2019_50021_MOESM1_ESM.docx]
